# Comments on Effects of routine physiotherapy with and without neuromobilization in the management of internal shoulder impingement syndrome: A randomized controlled trial

**DOI:** 10.12669/pjms.37.1.3880

**Published:** 2021

**Authors:** Rooma Rouf Chughtai, Farooq Azam Rathore

**Affiliations:** 1 Rooma Rouf Chughtai, 4^th^ Year DPT Student, Bahria University College of Physical Therapy, Karachi, Pakistan; 2 Farooq Azam Rathore, FCPS, MSc^,^ Department of Rehabilitation Medicine, PNS Shifa Hospital, Karachi, Pakistan

Dear Editor,

We have read the article “Effect of routine physiotherapy with and without neuromobilization in the management of internal shoulder impingement syndrome: A randomized controlled trial”^1^ by Akhtar et al with interest. Considering that the number of randomized clinical trials (RCT) from Pakistan is generally low, this is a good addition to the local biomedical literature. However, an RCT is considered the gold standard of clinical research and must adhere to the highest standards of conducting the trial and reporting. We recently reviewed this article in detail in our class as part of the Evidence based practice module. We used the Critical Appraisal Skills Programme (CASP) Randomised Controlled Trials Checklist (https://casp-uk.net/wp-content/uploads/2020/10/CASP_RCT_Checklist_PDF_Fillable_Form.pdf) for the critical appraisal and analysis of the article. While this article scored high in almost all questions, we were able to identify some issues which we feel are important and need some more clarification.

Authors stated that the baseline demographic characteristics of both groups were the same at the start of the trial. However, Table-I mentions only two parameters i.e. age and gender. There is a need to add more demographic characteristics in an RCT which can affect the outcomes. These may include duration of the disease, occupational status of the participant and the socioeconomic status. These are important parameters which must be reported. For example, the outcomes for a patient with less than four weeks duration of symptoms Vs. three months duration will likely be different. Similarly, outcomes in a housewife with shoulder impingement who must do household chores daily can differ from shoulder impingement in a banker with a sedentary lifestyle. Therefore, it is important that the base line demographics must include all important parameters which can affect the study outcomes.

The duration of study was from September 2016 to March 2018. However, as per the information provided the Institutional Review Board, University of Lahore gave the approval for the study after the completion of study. (IRB-UOL-FAHS/318/2018, dated: 24, April 2018). This needs to be explained as the ethics review committee/ Institutional board review approval must be obtained before the recruitment of first participant.

The trial was registered in the Iranian Registry of Clinical Trials with the identifier IRCT20190121042445N1. On examining the research protocol registered on the website (https://irct.ir/trial/37026) some discrepancies were noticed. The title of the registered protocol is “The Effects of Neuromobilization in Patients with Shoulder Impingement Syndrome on Pain, Strength, Range of Motion and Functional Disability Score” which is different from the title of the published manuscript. In addition, both pain, and range of motion (ROM) have been mentioned as the primary outcome measures. Secondary outcome measures include shoulder strength and functional disability score. However, in the manuscript authors have reported only one outcome measure i.e. Numeric Rating Scale for assessing outcomes of intervention. Improvement on one outcome measure cannot be considered as a proof of effectiveness when other outcome measure as mentioned in the registered research protocol have not been reported or mentioned in the manuscript.

In Pakistan sales of medicines is not well regulated and patients can buy all kind of medicines including pain killers from the pharmacies even without the prescription of physicians. There is a possibility that some patients in this study might be using pain killers in addition to the intervention and the improvement was due to the medicines rather than the intervention. We would like to know, how the authors address this major confounding factor of this trial?

Twenty-nine references have been cited in this manuscript and there is not even a single citation of a local article published from Pakistan. While we agree that there are no published manuscripts on the topic of neuromobilization in the management of internal shoulder impingement syndrome from Pakistan, many articles have been published on different forms and causes of shoulder impingement and the management strategies. At least some local manuscripts should have been cited.

We identified two minor mistakes of proof reading. March is misspelled as ‘Match” in the methodology section and instead of “socio-demographic” title of Table-II reads as “Scio-demographic”. Scrutiny of the galley proofs for minor errors and spelling mistakes is the responsibility of the authors.^2^

We submit these observations and comments as a critical appraisal of the manuscript rather than a critique of the excellent work performed by the authors.

## Response from the Author

1. We had selected chronic patients with more than three month symptoms duration. In this setting it was not possible to divide the patients in different occupation. In hospital setting all patients belong to almost similar socioeconomic status. These limitations were also mentioned in Ph.D. thesis Project.
2. IRB approval for the study was received on August 2, 2016 Ref. NO. IRB-UOL-FAHS/00238-II. It was ay mistake. Original letter of IRB approval has been uploaded on the OJS.
3. This manuscript was the part of Ph.D. thesis project. Other outcomes mentioned in Registry of Clinical Trials were used for other manuscripts of the thesis project, therefore only one outcome was used in this study.
4. In written informed consent form it was mentioned that patient will not take any medication and he/she will follow the principle.
5. There was no significant work on neuromobilization for shoulder impingent syndrome in local articles; therefore, no references were added from local articles published in Pakistan.
6. These two spelling mistakes occurred during proof reading. In the published manuscript in methodology section Match should be read as “March” and in the title of Table-II Scio-demographic should be considered as “socio-demographic”.
